# Online versus in-person training of community health workers to enhance hepatitis B virus screening among Korean Americans: Evaluating cost & outcomes

**DOI:** 10.1016/j.pmedr.2020.101131

**Published:** 2020-05-28

**Authors:** Theresa I. Shireman, Alexander C. Adia, Yin Tan, Lin Zhu, Joanne Rhee, Olorunseun O. Ogunwobi, Grace X. Ma

**Affiliations:** a Department of Health Services, Policy and Practice, School of Public Health, Brown University, Providence, RI, USA; b Center for Asian Health, Lewis Katz School of Medicine, Temple University, Philadelphia, PA, USA; c Department of Biological Sciences, Hunter College of the City University of New York, New York, NY, USA; d Department of Clinical Sciences, Lewis Katz School of Medicine, Temple University, Philadelphia, PA, USA

**Keywords:** Community health worker training, HBV screening and vaccination, Cost-effectiveness, Community-based participatory research, Korean Americans

## Abstract

From 2014 to 2018, we developed and implemented culturally appropriate interventions delivered by community health workers (CHWs) in Pennsylvania and New Jersey. To determine the most cost-effective approach, we recruited 40 predominantly foreign-born Korean American CHWs and used cluster sampling to assign them into two training groups (online training vs. in-person training). We prospectively assessed the cost of training 40 Korean American CHWs and the cost of subsequent HBV educational workshops delivered by the CHWs. We also assessed these costs relative to the success of each training approach in recruiting participants for HBV screening and vaccination. We found that the training costs per participant were higher for in-person training ($1.71 versus $1.12), while workshop costs per participant were lower for in-person training ($2.19 versus $4.22). Workshop attendee costs were comparable. After accounting for site clustering, there were no significant differences in total costs per participant ($24.55 for the online-trained group and $26.05 for the in-person group). In-person trained CHWs were able to generate higher HBV screening and vaccination rates (49.3% versus 21.4% and 17.0% versus 5.9%, respectively) among their participants compared with online-trained CHWs. Given better outcomes and no differences in costs, in-person training dominated the online training option. Despite the potential for efficiency to be gained with online training, CHWs who attended live training outperformed their online-trained colleagues. Elements of the didactic approach or practice with peers in the live session may have contributed to the superior training effectiveness and, ultimately, improved cost-effectiveness of the in-person approach.

## Highlights

• Training-costs/participant were higher for in-person training than online training.
• Workshop-costs/participant were lower for in-person training than online training.
• Total costs were higher for the online-trained group than the in-person group.
• In-person trained CHWs were more cost-effective than online-trained CHWs.
• CHWs who attended live training outperformed their online-trained colleagues.

## 1 Introduction

In the United States, approximately 2.2 million people live with chronic hepatitis B Virus (HBV) infection (Centers for Disease Control and Prevention, 2019). Asians Americans and Pacific Islanders are disproportionately affected, with foreign-born Asian immigrants in particular bearing the highest prevalence of chronic HBV (Kowdley et al., 2012). HBV infection is a significant risk factor for liver cancer. Among Asian Americans, high chronic HBV prevalence contributes to disparities in liver cancer incidence and mortality rates (Miller et al., 2008, Thompson et al., 2016). Korean Americans, which make up the fifth-largest Asian-American subgroup in the United States, have the second highest rate of liver cancer and the highest liver cancer-related mortality rate of Asian Americans (Huang et al., 2013). These high incidence and mortality rates for liver cancer are driven by a combination of poor awareness, low screening rates, and under-vaccination for HBV in Korean-American populations (Bastani et al., 2007, Ma et al., 2012, Misra et al., 2013, Strong et al., 2012, Bastani et al., 2015).

Early detection and care for chronic HBV can prevent deaths from liver cancer and can prevent costly implications for both patients and healthcare systems (Post et al., 2011). In fact, programs to improve screening and vaccination for HBV among Asian and Pacific Islander adults have been shown to be cost-effective (Hutton et al., 2007). In the case of Korean Americans, however, HBV programs need to address complex challenges, including overcoming barriers in access to screening and vaccination and to health care generally. Programs must also be prepared to address a lack of awareness and understanding regarding HBV and liver cancer, as well as be able to help participants overcome fears and concerns about screening and the possibility of a positive result. One approach to address these issues is to adopt the community-based participatory research (CBPR) approach which can yield culturally appropriate interventions tailored to specific issues that Korean Americans regularly face (Israel et al., 1998, Ma et al., 2012). Previous attempts to combat HBV disparities among Asian Americans have included city-wide efforts in New York City and San Francisco. These programs focused on improving HBV screening and testing and were led by coalitions of community members alongside other key stakeholders. The success of these efforts has played a key role in demonstrating the value of community-focused HBV screening campaigns (Bailey et al., 2011, Pollack et al., 2011) .

Community health workers (CHWs) have become an increasingly important part of implementing interventions designed to address health disparities, especially among minority populations (Malcarney et al., 2017). While studies have found that CHWs played significant roles in promoting preventive health behaviors in Korean Americans (Han et al., 2016, Islam et al., 2017, Kim et al., 2016), few have fully evaluated the cost-effectiveness of CHWs (Viswanathan et al., 2010). Only one previous study examined the cost-effectiveness of CHWs to promote breast and cervical cancer screening among Korean Americans and the researchers found the CHW-led intervention offered a more cost-effective approach for cancer screening than did other programs (Schuster et al., 2015). Training CHWs takes time and can consume resources when workers are not paid (e.g., opportunity costs). The most cost-effective approach to training, however, remains unknown. CHWs may be trained to conduct their interventions through a variety of approaches, though historically they have been trained in a live group setting. Recent evidence has supported the effectiveness of online training in nursing (Du et al., 2013, McCutcheon et al., 2015), social work (Phelan, 2015), and provision of homeless services (Olivet et al., 2016),^1^ and comparative advantages may include increased convenience and decreased costs (Ruggeri et al., 2013). It remains an open question as to whether one approach to training CHWs leads to better intervention effectiveness and, consequently, how training modality affects the cost of achieving the targeted behavior.

To address this gap, we investigated the cost-effectiveness (Haddix et al., 2003) of a community-based, multi-level intervention for HBV screening and vaccination implemented in Korean churches in Pennsylvania and New Jersey. Specifically, we evaluated, a) the costs for two different CHW training approaches (online versus in-person training programs), and b) the screening and vaccination rates for HBV among community members participating in an intervention conducted by the aforementioned CHWs. Insights from this study proved useful to those considering the most efficient way to implement interventions through CHWs, especially when supported with limited funding.

## 2 Methods

### 2.1 Study design

From 2014 to 2018, we conducted a prospective cost analysis of an intervention developed through a CBPR approach. The purpose of the intervention was to increase HBV screening and vaccination rates among Korean American communities. The intervention relied upon CHWs to learn about HBV and to train fellow church members about the need for screening and vaccination. Although CHWs were volunteers (unpaid), we identified and valued their time. Furthermore, we assigned time costs for the workshop participants in accordance with a societal perspective.

### 2.2 Procedures

In this CBPR study, community members, health-care providers, and members from Korean churches helped guide the planning and development of recruitment strategies and the culturally appropriate HBV intervention for implementation in various Korean churches serving predominantly foreign-born congregations. We selected the church settings because previous studies have shown that faith-based organizations serve a significant role in the implementation and dissemination of cancer-related screening intervention in Korean American communities (Fang et al., 2017, Han et al., 2016, Ma et al., 2018, Ma et al., 2012). A total of 20 Korean churches in Pennsylvania and New Jersey were enrolled as research sites. We used cluster random sampling to assign 10 sites (20 CHWs) to the in-person CHW training group and 10 sites (20 CHWs) to the online CHW training group. In each church, we recruited two CHWs. The CHWs were recommended by the pastors and leaders from the participating Korean churches. The eligibility criteria of CHWs included 1) age 21 or above, 2) fluent speaking and written proficiency of Korean language, and at least intermediate proficiency of English language, 3) high school graduation education level or above, 4) basic computer skills with internet access, 5) membership to one of the participating Korean church, and 6) willingness to serve congregants of the participating Korean churches. When CHW were recruited, they did not know which type of training they would receive.

CHWs in both online and in-person training groups were provided three training sessions including topics, including (1) “Getting Started,” an overview of the Hepatitis B Intervention program, (2) “How to implement Hepatitis B Intervention Program,” and (3) “How to Use Program Materials and Tools for Implementing the Intervention Project. CHWs in in-person group received two full-day training by experienced bilingual research team members at each enrolled church. A CHW’s guide binder with both hard copy and E-copy of materials and tools/forms for implementing the project was provided to the CHWs. In-person training involved dynamic training approaches, presentations, discussions, role play activities of workshop delivery, research data collection, practice of patient navigation assistance, and using project materials and evaluation tools. CHWs in the online group received a user instruction for online training platform via email. The CHWs also received assigned username and password to log in the online platform. CHW could choose either English or Korean language to review and listen. The platform included three sub-pages, (1) three training session modules covering the topics described above, (2) downloadable project implementation materials and tools/forms for CHWs to be used in filed, and (3) implementation technical assistance tools/materials for CHWs.

CHWs in both groups implemented the project with three major roles: recruiting participants from their affiliated church, delivering community education workshops and providing patient navigation for participants’ need on screening or vaccination after the education workshops. Each of the 40 CHW was asked to recruit community members to participate in educational workshops on HBV screening and vaccination. The CHWs worked with research staff adopted a variety of recruitment strategies in the churches to generate awareness of the study, and then performed eligibility screening and obtained informed consent among eligible individuals who expressed interest in participating in the study. Inclusion criteria of participating in the study were: 1) self-identified Korean ethnicity, 2) ages 18 and above, 3) willingness to participate in study, 4) accessible by telephone (for scheduling and follow up purpose), 5) presence in the same study geographic area for a period of one year (to minimize participant attrition), 6) not enrolled in any HBV intervention (to prevent a potential program impact), 7) never had HBV screening, and 8) not been diagnosed with HBV or liver disease complication condition or liver cancer (the intervention was designed only for increasing HBV screening and vaccination). Trained CHWs delivered educational sessions. In total, 1,255 community members (588 from the in-person CHW training group and 667 from the online CHW training group) participated in the educational workshops among which 1,044 (481 from the in-person CHW training group and 523 from the online CHW training group) participated the baseline and post-intervention survey. The survey completion rate was 80% overall, 82% for the in-person CHW training group, and 78% for the online CHW training group.

All research team members, the community coordinator, and designated church staff and CHWs received intensive training regarding a study protocol, facilitation guidelines, and participants' rights by the study principal investigator and co investigators before the study began. All participants provided written consent. The study was approved by the institutional review board (IRB) of Temple University.

### 2.3 Measures

Information regarding age, sex, education, marital status, household income, employment, health insurance, regular physician status, ability to speak English, and English proficiency was collected at baseline for each church member participant. Information regarding age, sex, education, marital status, English proficiency, and computer use and proficiency were also collected from each CHW at baseline. Survey questionnaires were developed in English, translated into Korean, and back translated into English by bilingual translators to ensure accuracy and cultural competency.

We assessed HBV screening at 6- and 12-month follow-ups. Bilingual interviewers contacted participants at the 6‐month follow‐up and asked participants whether they received HBV screening with the response options of “yes” or “no.” Vaccination status among participants who were screened for having no HBV immunity also was measured. Specifically, participants were asked to report whether and how many times they received vaccination against HBV infection at the 12‐month follow‐up after the intervention. With permission, we also confirmed the self-reported blood test for HBV screening and vaccinations with each participant’s health-care provider. For the effectiveness analyses, we used the 12-month screening and vaccination rates.

The relative costs of the two approaches (in-person training versus online training) in disseminating the intervention was assessed from a societal perspective using generally accepted principles for public-health interventions.^2^ We included time-based costs for CHWs and workshop attendees but did not include direct medical costs for HBV screening and vaccination, making our approach a modified societal perspective.

Though CHWs were not paid for their involvement, we estimated the value of their time. First, we calculated the length of time CHWs to participated in each training approach. For in-person training, members of the project team prospectively documented live training session time. For the on-line training group, CHWs were asked to self-report the amount of time it took for them to review the materials until they passed the evaluation form. Intervention workshops with church members generally lasted one hour, regardless of the mode of CHW training, but the CHWs conducted different numbers of workshops with varying numbers of workshop attendees. This created differential workshop (intervention) time costs for the CHWs. We did not include CHW time spent recruiting workshop participants or the research staff time spent providing the in-person training or monitoring the on-line training. To determine CHW costs of participating in the training and leading the workshops, we applied 2014 national, age- and gender-adjusted median weekly earnings for all workers from the Bureau of Labor Statistics (www.bls.gov) with conversion to hourly rates.

We included the value of workshop attendees’ time based assuming a one-hour session. As with the CHWs, we assigned age- and gender-adjusted wages from the BLS. In cases where age or gender were missing, we assigned an estimated hourly wage from their self-reported income level. Finally, workshop materials and their development were identical across the treatment arms, and thus, no costs were assigned for these components of the intervention.

Costs were valued in 2014 dollars, to coincide with time at study enrollment.

### 2.4 Analyses

Comparisons between CHWs and workshop attendees were tested with cluster-adjusted t-tests for continuous measures or cluster-adjusted chi-square tests for nominal or categorical variables. We used the “cltest” package to conduct all cluster-adjusted analyses (Herrin, 2012). To test for cost and outcome differences between the in-person and on-line trained CHWs, we used multilevel, mixed models to account for study site clustering and adjusting for age and gender. For costs, we used a linear mixed model and for vaccination and screening, we used binary logistic regression. Analyses were conducted using IBM SPSS 25.

## 3 Results

As was shown in Table 1, CHWs were on average around 47 years old, with slightly more males than females. They were predominately foreign-born and had lived in the United States for approximately 17 years on average. All had completed high school, and just under half were employed. CHWs’ English proficiency was limited, though the majority of CHWs used a computer daily and had good self-rated computer skills. There were no significant differences in baseline characteristics between the CHWs who trained online as compared to those who attended a live training session. The imputed age- and gender-adjusted mean hourly wages for CHWs were also not significantly different ($20.73 and $20.84, respectively).

**Table 1:** **. Descriptive characteristics of Community Health Workers** (From 2014 to 2018 in Pennsylvania and New Jersey).

	All	In-person (n = 20)	Online (n = 20).	*P* value
Age in years, mean (SD)	46.8 (13.4)	48.7 (13.2)	44.9 (13.7)	0.46
Gender, *n* (%)				0.34
Female	19 (47.5%)	11 (55.0%)	8 (40.0%)	
Male	21 (52.5%)	9 (45.0%)	12 (60.0%)	
US-born, *n* (%)				0.29
Yes	2 (5.0%)	0 (0.0%)	2 (10.0%)	
No	38 (95.0%)	20 (100.0%)	18 (90.0%)	
Years in the U.S., mean years (SD)	16.7 (9.4)	16.2 (10.7)	17.1 (8.3)	0.78
Marital status, *n* (%)				0.58
Married	30 (75.0%)	14 (70.0%)	16 (80.0%)	
Single/divorced/separated/widowed	10 (25.0%)	6 (30.0%)	4 (20.0%)	
Education, *n* (%)				0.43
High school or below	6 (15.0%)	4 (20.0%)	2 (10.0%)	
College or above	34 (86.0%)	16 (80.0%)	18 (90.0%)	
Employment status, *n* (%)				0.78
Employed	17 (42.5%)	9 (45.0%)	8 (40.0%)	
Unemployed or not in labor force	23 (57.5%)	11 (55.0%)	12 (60.0%)	
English proficiency, *n* (%)				n/a
Not at all or not well	20 (50.0%)	10 (50.0%)	10 (50.0%)	
Well or very well	20 (50.0%)	10 (50.0%)	10 (50.0%)	
Frequency of computer/internet use, *n* (%)				0.17
Some days or often	12 (30.0%)	8 (40.0%)	4 (20.0%)	
Almost everyday	28 (70.0%)	12 (60.0%)	16 (80.0%)	
Computer skills, *n* (%)				0.18
Not well	14 (35.0%)	9 (45.0%)	5 (25.0%)	
Well or very well	26 (65.0%)	11 (55.0%)	15 (75.0%)	
Mean Hourly Wage, Imputed	$20.79	$20.73	$20.84	

As presented in Table 2, the educational workshop participants had an average age of 46.2, with no significant difference between the two study arms. While there was a higher proportion of women participants in the in-person training group than in the on-line group (57.8% versus 50.8%), the difference was not statistically significant (p = 23). There were no differences in the proportion of foreign-born participants or in participants’ length of time in the United States. No other significant differences in demographic characteristics were observed between the two participant groups.

**Table 2:** **. Descriptive characteristics workshop attendees** (From 2014 to 2018 in Pennsylvania and New Jersey).

	Training Group Participants			
	All	In-person (n = 481)	On-line (n = 523)	*P* value
Age in years, mean (SD)	46.2 (14.41)	45.2 (14.54)	47.2 (14.23)	0.59
Gender, *n* (%)				0.23
Female	537 (54.1%)	274 (57.8%)	263 (50.8%)	
Male	455 (45.9%)	200 (42.2%)	255 (49.2%)	
US-born, *n* (%)				0.57
Yes	79 (8.0%)	32 (6.7%)	47 (9.2%)	
No	909 (92.0%)	443 (93.3%)	466 (90.8%)	
Years in the U.S., mean years (SD)	16.8 (10.6)	16.6 (11.1)	17.0 (10.1)	0.84
Marital status, *n* (%)				0.90
Married	706 (71.3%)	333 (70.7%)	373 (71.9%)	
Single/divorced/separated/widowed	284 (28.7%)	138 (29.3%)	146 (28.1%)	
Education, *n* (%)				0.56
High school or below	314 (32.2%)	160 (34.3%)	154 (30.3%)	
College or above	661 (67.8%)	307 (65.7%)	354 (69.7%)	
Employment status, *n* (%)				0.76
Employed	552 (55.7%)	259 (54.2%)	293 (57.1%)	
Unemployed or not in labor force	439 (4.3%)	219 (45.8%)	220 (42.9%)	
Annual household income, *n* (%)				0.80
Less than $20,000	254 (28.8%)	126 (29.7%)	128 (28.0%)	
$20,000 or above	629 (71.2%)	299 (70.3%)	330 (72.0%)	
Have health insurance, *n* (%)				0.58
Yes	605 (60.9%)	279 (58.4%)	326 (63.2%)	
No	389 (39.1%)	199 (41.6%)	190 (36.8%)	
Have regular physician				0.79
Yes	532 (54.5%)	251 (53.2%)	281 (55.6%)	
No	446 (45.5%)	221 (46.8%)	224 (44.4%)	
English proficiency				0.94
Not at all or not well	531 (53.1%)	256 (53.5%)	275 (52.9%)	
Well or very well	468 (46.8%)	223 (46.5%)	245 (47.1%)	

Initially, for the intervention, 1,004 Korean American community members were recruited by CHWs and received education workshops: 481 participants were allocated to in-person trained CHWs, and 523 participants completed baseline activities. Overall, 73.8% of community members recruited and educated by in-person trained CHWs completed all study procedures compared to 69.4% of those recruited and educated from online trained CHWs.

Training and workshop costs were comparable between the two training approaches (Table 3). The live training sessions generally lasted two hours, while the online training group reported spending less than one and a half hours (82.5 min) working through the materials and evaluation process. This shortened training time produced a lower mean cost of training and total cost of training for the online group. The number of workshop participants recruited per church ranged from 42 to 56 (mean 50.2, SD 3.4) and workshops generally lasted one hour. Sites varied in the number of participants attending each workshop (range 1–24, mean 8.5, SD 7.5).

**Table 3:** **Relative costs (in 2014 dollars) and effectiveness of CHW training approaches (**From 2014 to 2018 in Pennsylvania and New Jersey**)**.

	In-person training group	On-line training group
Per participant costs:	(CHW n = 20)	(CWH n = 20)
CHW training costs ($), mean ± std dev	1.71 ± 0.434	1.12 ± 0.297
CHW workshop costs ($), mean ± SD	2.19 ± 1.50	4.22 ± 3.41
Attendee workshop costs ($), mean ± SD	20.51 ± 4.69	20.75 ± 4.20
Total costs ($), mean ± SD	24.45 ± 4.56	26.05 ± 5.06
Outcomes:	(participant n = 481)	(participant n = 523)
Received HBV screening	49.3%	21.4%
Received HBV vaccination	17.0%	5.9%

Attendee and CHW time costs based on BLS age- and gender-wage rates for 2014; std dev, standard deviation.

After applying CHW hourly wage rates to training and workshop time, CHW costs per participant differed by training arm (Table 3). Training costs per participant for in-person versus online training averaged $1.71 versus $1.12, and workshop costs per participant for in-person versus online training averaged $2.19 versus $4.22. The cost difference between groups was driven by smaller, more frequent workshops given by the online-trained CHWs (112 workshops) as compared to the in-person-trained CHWs (57 workshops); in-person-trained CHWs held workshops with an average of 7.54 participants compared to an average of 4.85 participants in workshops run by online-trained CHWs. Workshop attendee costs were comparable ($20.51 versus $20.75). Site specific costs ranged from $20.04 to $33.17. After accounting for clustering, total costs per participant did not differ between the CHW training arms (p = 0.085).

With respect to outcomes, HBV screening rates were higher for participants who attended intervention with in-person trained CHWs (49.3%) as compared with those who attended intervention with online trained CHWs (21.4%). At 12-month follow-up, vaccination rates were also higher for participants who attended in-person trained CHW sessions (17.0%) as compared with online-trained CHW sessions (5.9%). After adjusting for site clustering, these differences were significantly different: HBV screening rates were significant higher (p = 0.014) for the in-person trained CHW group and HBV vaccination rates were also significantly higher (p = 0.029).

## 4 Discussion

To our knowledge, this is one of the first cost and outcome evaluations of online-trained versus in-person-trained CHWs. In this study, preparing CHWs via in-person training took longer and was slightly more expensive than training CHWs through an online program. However, participants who attended workshops run by in-person-trained CHWs were more likely to receive screening and vaccination for HBV. Our analyses show that although it may cost less to train CHWs through an online program, in-person training was more cost-effective in promoting HBV screening and vaccination among Korean Americans.

The observed differences may be driven by the inclusion of peers in training for the in-person approach; such inclusion did not occur with online training. Inclusion of peers allows for troubleshooting of issues present in group dynamics and may have improved comfort in handling larger groups among in-person trained CHWs. Indeed, online-trained CHWs ran nearly double the number of workshops that in-person trained CHWs did, suggesting that in-person-trained CHWs may have been more comfortable in navigating participant education in a way that directly translated to better outcomes. The number of participants within a workshop might have also played a role in decision-making of the participants. The average size of the workshops held by the in-person-trained CHWs was bigger than that of the ones held by the online-trained CHWs. It is possible that the bigger size of the workshops allowed participants more opportunities to interact with and motivate each other to seek out screening and vaccination. Future research comparing online and in-person training for CHWs could implement a mixed-methods design that integrates a qualitative component for both CHWs and participants. Doing so may enable a richer understanding of differences in the way CHWs in each training approach conduct study activities and may improve interpretation of the quantitative findings.

Nonetheless, online training is a convenient method for CHWs training that has little geographic and time limitations. Future online training should better facilitate the interactions among CHWs and seek to improve CHW’s skills in building rapport with participants and facilitating discussion among participants.

These findings conflict with previous findings in which online-trained CHWs produced similar health outcomes as in-person-trained CHWs for an intervention designed to improve cancer screening for attendees of African-American churches (Holt et al., 2018). Another study (Clement et al., 2012) compared filmed versus live social contact intervention to reduce mental health stigma in student nurses and found that filmed social contact intervention was more cost-effective, which contradicts our study. One explanation might be that since the HBV screening and vaccination rates were low among Korean Americans, the knowledge and behavior change gap that CHWs in our study had to help community members overcome might have been substantively higher. This factor, together with skills for navigating group dynamics that were acquired in workshops by in-person-trained CHWs but not online-trained CHWs, may have contributed to the differences observed in this study.

Regardless of the CHW training group, the HBV screening rates among participants in this study were much higher than the rates reported in previous studies of CHW-led HBV screening interventions in Cambodian Americans (Taylor et al., 2013) and Chinese Americans or Canadians (Taylor et al., 2009). The vaccination rates reported in our study was lower than the rate (46.03%) in a study of CHW-led HBV intervention among an aggregate of four Asian ethnic groups (Lee, 2018).

There are a few limitations worth noting. First, the majority of participants were foreign-born Korean adults in Pennsylvania and New Jersey. As such, these findings may not be generalizable for CHWs working with communities of Korean Americans or other Korean adults in different geographical areas of the United States. Second, despite the cluster randomization procedure used, participants educated by online versus in-person CHWs differed based on age and sex. It is also likely that if this study were to be scaled-up, costs and outcomes may be different from those observed for this study. Furthermore, we used publicly available age- and gender-adjusted national wage rates, which may not be directly applicable to Korean CHWs. Future cost-effectiveness analyses for studies targeting larger samples of Korean adults are needed.

Despite the potential for efficiency gained with online training, CHWs who attend live training outperformed their online-trained colleagues. Elements of the didactic approach or practice with peers in the live session may have led to the edge in effectiveness. This result can be used to inform the development of future implementation research on HBV screening among underserved minority populations. Given that foreign-born Asian immigrants make up 58% of the total cases of HBV infection in the US (Centers for Disease Control and Prevention, 2015), information on effective ways of HBV intervention implementation are urgently needed to promote HBV screening in this health disparity community. This study responds to the needs. It is an initial step to a large-scale study to assess the cost-effectiveness of CHW-led intervention in various Asian American communities with high HBV burden.

## CRediT authorship contribution statement

**Theresa I. Shireman:** Methodology, Formal analysis, Writing - review & editing. **Alexander C. Adia:** Writing - original draft. **Yin Tan:** Project administration, Writing - review & editing. **Lin Zhu:** Formal analysis, Writing - original draft, Writing - review & editing. **Joanne Rhee:** . **Olorunseun O. Ogunwobi:** Methodology, Writing - review & editing. **Grace X. Ma:** Funding acquisition, Supervision, Writing - review & editing.

## Declaration of Competing Interest

The authors declare that they have no known competing financial interests or personal relationships that could have appeared to influence the work reported in this paper.

## References

[b0005] Bailey M.B., Shiau R., Zola J., Fernyak S.E., Fang T., So S.K.S., Chang E.T. (2011). San Francisco hep B free: a grassroots community coalition to prevent hepatitis B and liver cancer. J Community Health.

[b0010] Bastani, R., Glenn, B.A., Maxwell, A.E., Jo, A.M., Herrmann, A.K., Crespi, C.M., Wong, W.K., Chang, L.C., Stewart, S.L., Nguyen, T.T., Chen, M.S., Taylor, V.M., 2015. Cluster-Randomized Trial to Increase Hepatitis B Testing among Koreans in Los Angeles. Cancer Epidemiol Biomarkers Prev cebp.1396.2014. https://doi.org/10.1158/1055-9965.EPI-14-1396. 10.1158/1055-9965.EPI-14-1396 PMC4560609 26104909

[b0015] Bastani R., Glenn B.A., Maxwell A.E., Jo A.M. (2007). Hepatitis B testing for liver cancer control among Korean Americans. Ethn. Dis..

[b0020] Centers for Disease Control and Prevention, 2019. U.S. 2016 Surveillance Data for Viral Hepatitis [WWW Document]. URL https://www.cdc.gov/hepatitis/statistics/2016surveillance/index.htm (accessed 3.2.19).

[b0025] Centers for Disease Control and Prevention, 2015. Viral Hepatitis - CDC Recommendations for Specific Populations and Settings: Asian and Pacific Islanders [WWW Document]. URL http://www.cdc.gov/hepatitis/populations/api.htm (accessed 4.7.16).

[b0030] Clement S., van Nieuwenhuizen A., Kassam A., Flach C., Lazarus A., Castro M.D., McCrone P., Norman I., Thornicroft G. (2012). Filmed v. live social contact interventions to reduce stigma: randomised controlled trial. Brit. J. Psych..

[b0035] Du S., Liu Z., Liu S., Yin H., Xu G., Zhang H., Wang A. (2013). Web-based distance learning for nurse education: a systematic review. Int Nurs Rev.

[b0040] Fang C.Y., Ma G.X., Handorf E.A., Feng Z., Tan Y., Rhee J., Miller S.M., Kim C., Koh H.S. (2017). Addressing multilevel barriers to cervical cancer screening in Korean American women: A randomized trial of a community-based intervention. Cancer.

[b0045] Haddix A.C., Teutsch S.M., Corso P.S. (2003). Prevention Effectiveness: A Guide to Decision Analysis and Economic Evaluation.

[b0050] Han H.-R., Song Y., Kim M., Hedlin H.K., Kim K., Ben Lee H., Roter D. (2016). Breast and Cervical Cancer Screening Literacy Among Korean American Women: A Community Health Worker-Led Intervention. Am. J. Public Health.

[b0055] Herrin J. CLTEST: Stata Modules for Performing Cluster-Adjusted Chi-Square and t-Tests. Statistical Software Components S424901, Boston College Department of Economics, revised 03 Feb 2012. Accessed May 10, 2020. https://ideas.repec.org/c/boc/bocode/s424901.html.

[b0060] Holt C.L., Tagai E.K., Santos S.L.Z., Scheirer M.A., Bowie J., Haider M., Slade J. (2018). Web-based versus in-person methods for training lay community health advisors to implement health promotion workshops: participant outcomes from a cluster-randomized trial. Transl. Behav. Med..

[b0065] Huang, V., Li, W., Tsai, J., Begier, E., 2013. Cancer Mortality among Asians and Pacific Islanders in New York City, 2001–2010 [WWW Document]. J. Cancer Epidemiol. URL https://www.hindawi.com/journals/jce/2013/986408/ (accessed 2.28.19). 10.1155/2013/986408 PMC3876672 24454374

[b0070] Hutton D.W., Tan D., So S.K., Brandeau M.L. (2007). Cost-effectiveness of screening and vaccinating Asian and Pacific Islander adults for hepatitis B. Ann. Intern. Med..

[b0075] IBM Corp, 2017. IBM SPSS Statistics for Windows, Version 25.0. Armonk, NY.

[b0080] Islam N., Shapiro E., Wyatt L., Riley L., Zanowiak J., Ursua R., Trinh-Shevrin C. (2017). Evaluating community health workers’ attributes, roles, and pathways of action in immigrant communities. Prev. Med..

[b0085] Israel B.A., Schulz A.J., Parker E.A., Becker A.B. (1998). Review of community-based research: assessing partnership approaches to improve public health. Annu Rev Public Health.

[b0090] Kim K.B., Kim M.T., Lee H.B., Nguyen T., Bone L.R., Levine D. (2016). Community Health Workers Versus Nurses as Counselors or Case Managers in a Self-Help Diabetes Management Program. Am. J. Public Health.

[b0095] Kowdley K.V., Wang C.C., Welch S., Roberts H., Brosgart C.L. (2012). Prevalence of chronic hepatitis B among foreign-born persons living in the United States by country of origin. Hepatology.

[b0100] Lee J. (2018). Active Navigation in a Hepatitis B Program: Screening, Vaccination and Patient Assistance for Asian Americans in Michigan. Arch. Hepat. Res..

[b0105] Ma G.X., Gao W., Tan Y., Chae W.G., Rhee J. (2012). A community-based participatory approach to a hepatitis B intervention for Korean Americans. Prog. Community Health Partnersh..

[b0110] Ma, G.X., Lee, M.M., Tan, Y., Hanlon, A.L., Feng, Z., Shireman, T.I., Rhee, J., Wei, Z., Wong, F., Koh, H.S., Kim, C., York, W., 2018. Efficacy of a community-based participatory and multilevel intervention to enhance hepatitis B virus screening and vaccination in underserved Korean Americans. Cancer n/a–n/a. https://doi.org/10.1002/cncr.31134. 10.1002/cncr.31134 PMC5821582 29131316

[b0115] Malcarney M., Pittman P., Quigley L., Horton K., Seiler N. (2017). The Changing Roles of Community Health Workers. Health Serv Res.

[b0120] McCutcheon K., Lohan M., Traynor M., Martin D. (2015). A systematic review evaluating the impact of online or blended learning vs. face-to-face learning of clinical skills in undergraduate nurse education. J Adv Nurs.

[b0125] Miller B.A., Chu K.C., Hankey B.F., Ries L.A.G. (2008). Cancer incidence and mortality patterns among specific Asian and Pacific Islander populations in the U.S. Cancer Causes Control.

[b0130] Misra R., Jiobu K., Zhang J., Liu Q., Li F., Kirkpatrick R., Ho J. (2013). Racial Disparities in Hepatitis B Infection in Ohio: Screening and Immunization Are Critical for Early Clinical Management. J. Invest. Med..

[b0135] Olivet J., Zerger S., Greene R.N., Kenney R.R., Herman D.B. (2016). Online Versus Face-To-Face Training of Critical Time Intervention: A Matching Cluster Randomized Trial. Am J Distance Educ.

[b0140] Phelan J.E. (2015). The Use of E-Learning in Social Work Education. Soc Work.

[b0145] Pollack H., Wang S., Wyatt L., Peng C., Wan K., Trinh-Shevrin C., Chun K., Tsang T., Kwon S. (2011). A Comprehensive Screening And Treatment Model For Reducing Disparities In Hepatitis B. Health Aff..

[b0150] Post S.E., Sodhi N.K., Peng C., Wan K., Pollack H.J. (2011). Simulation Shows That Early Treatment of Chronic Hepatitis B Infection Can Cut Deaths and Be Cost-Effective. Health Aff (Millwood).

[b0155] Ruggeri K., Farrington C., Brayne C. (2013). A Global Model for Effective Use and Evaluation of e-Learning in Health. Telemed J E Health.

[b0160] Schuster A.L., Frick K.D., Huh B.-Y., Kim K.B., Kim M., Han H.-R. (2015). Economic evaluation of a community health worker-led health literacy intervention to promote cancer screening among Korean American women. J Health Care Poor Underserved.

[b0165] Strong C., Lee S., Tanaka M., Juon H.-S. (2012). Ethnic differences in prevalence and barriers of HBV screening and vaccination among Asian Americans. J. Community Health.

[b0170] Taylor V.M., Bastani R., Burke N., Talbot J., Sos C., Liu Q., Do H., Jackson J.C., Yasui Y. (2013). Evaluation of a Hepatitis B Lay Health Worker Intervention for Cambodian Americans. J. Commun. Health.

[b0175] Taylor V.M., Hislop T.G., Tu S.-P., Teh C., Acorda E., Yip M.-P., Woodall E., Yasui Y. (2009). Evaluation of a Hepatitis B Lay Health Worker Intervention for Chinese Americans and Canadians. J Commun. Health.

[b0180] Thompson C.A., Gomez S.L., Hastings K.G., Kapphahn K., Yu P., Shariff-Marco S., Bhatt A.S., Wakelee H.A., Patel M.I., Cullen M.R., Palaniappan L.P. (2016). The Burden of Cancer in Asian Americans: A Report of National Mortality Trends by Asian Ethnicity. Cancer Epidemiol. Biomarkers Prev..

[b0185] Viswanathan M., Kraschnewski J.L., Nishikawa B., Morgan L.C., Honeycutt A.A., Thieda P., Lohr K.N., Jonas D.E. (2010). Outcomes and Costs of Community Health Worker Interventions: A Systematic Review. Med. Care.

